# Immune rebalancing at the maternal-fetal interface of maternal SARS-CoV-2 infection during early pregnancy

**DOI:** 10.1093/procel/pwae006

**Published:** 2024-03-05

**Authors:** Chenxiang Xi, Zihui Yan, Dandan Bai, Yalin Zhang, Beiying Wang, Xiaoxiao Han, Li Wu, Xiaohui Shi, Zhiyi Hu, Ming Tang, Zhongqu Su, Yingdong Liu, Binya Liu, Jiqing Yin, Hong Wang, Xiaocui Li, Yanping Zhang, Shaorong Gao, Wenqiang Liu

**Affiliations:** Shanghai Key Laboratory of Maternal Fetal Medicine, Clinical and Translational Research Center of Shanghai First Maternity and Infant Hospital, Shanghai Institute of Maternal-Fetal Medicine and Gynecologic Oncology, Frontier Science Center for Stem Cell Research, School of Life Sciences and Technology, Tongji University, Shanghai 200092, China; Shanghai Key Laboratory of Maternal Fetal Medicine, Clinical and Translational Research Center of Shanghai First Maternity and Infant Hospital, Shanghai Institute of Maternal-Fetal Medicine and Gynecologic Oncology, Frontier Science Center for Stem Cell Research, School of Life Sciences and Technology, Tongji University, Shanghai 200092, China; Shanghai Key Laboratory of Maternal Fetal Medicine, Clinical and Translational Research Center of Shanghai First Maternity and Infant Hospital, Shanghai Institute of Maternal-Fetal Medicine and Gynecologic Oncology, Frontier Science Center for Stem Cell Research, School of Life Sciences and Technology, Tongji University, Shanghai 200092, China; Jiaxing Maternity and Child Health Care Hospital, Jiaxing 314050, China; Shanghai Key Laboratory of Maternal Fetal Medicine, Clinical and Translational Research Center of Shanghai First Maternity and Infant Hospital, Shanghai Institute of Maternal-Fetal Medicine and Gynecologic Oncology, Frontier Science Center for Stem Cell Research, School of Life Sciences and Technology, Tongji University, Shanghai 200092, China; Shanghai Key Laboratory of Maternal Fetal Medicine, Clinical and Translational Research Center of Shanghai First Maternity and Infant Hospital, Shanghai Institute of Maternal-Fetal Medicine and Gynecologic Oncology, Frontier Science Center for Stem Cell Research, School of Life Sciences and Technology, Tongji University, Shanghai 200092, China; Shanghai Key Laboratory of Maternal Fetal Medicine, Clinical and Translational Research Center of Shanghai First Maternity and Infant Hospital, Shanghai Institute of Maternal-Fetal Medicine and Gynecologic Oncology, Frontier Science Center for Stem Cell Research, School of Life Sciences and Technology, Tongji University, Shanghai 200092, China; Shanghai Key Laboratory of Maternal Fetal Medicine, Clinical and Translational Research Center of Shanghai First Maternity and Infant Hospital, Shanghai Institute of Maternal-Fetal Medicine and Gynecologic Oncology, Frontier Science Center for Stem Cell Research, School of Life Sciences and Technology, Tongji University, Shanghai 200092, China; Shanghai Key Laboratory of Maternal Fetal Medicine, Clinical and Translational Research Center of Shanghai First Maternity and Infant Hospital, Shanghai Institute of Maternal-Fetal Medicine and Gynecologic Oncology, Frontier Science Center for Stem Cell Research, School of Life Sciences and Technology, Tongji University, Shanghai 200092, China; Shanghai Key Laboratory of Maternal Fetal Medicine, Clinical and Translational Research Center of Shanghai First Maternity and Infant Hospital, Shanghai Institute of Maternal-Fetal Medicine and Gynecologic Oncology, Frontier Science Center for Stem Cell Research, School of Life Sciences and Technology, Tongji University, Shanghai 200092, China; Shanghai Key Laboratory of Maternal Fetal Medicine, Clinical and Translational Research Center of Shanghai First Maternity and Infant Hospital, Shanghai Institute of Maternal-Fetal Medicine and Gynecologic Oncology, Frontier Science Center for Stem Cell Research, School of Life Sciences and Technology, Tongji University, Shanghai 200092, China; Shanghai Key Laboratory of Maternal Fetal Medicine, Clinical and Translational Research Center of Shanghai First Maternity and Infant Hospital, Shanghai Institute of Maternal-Fetal Medicine and Gynecologic Oncology, Frontier Science Center for Stem Cell Research, School of Life Sciences and Technology, Tongji University, Shanghai 200092, China; Shanghai Key Laboratory of Maternal Fetal Medicine, Clinical and Translational Research Center of Shanghai First Maternity and Infant Hospital, Shanghai Institute of Maternal-Fetal Medicine and Gynecologic Oncology, Frontier Science Center for Stem Cell Research, School of Life Sciences and Technology, Tongji University, Shanghai 200092, China; Shanghai Key Laboratory of Maternal Fetal Medicine, Clinical and Translational Research Center of Shanghai First Maternity and Infant Hospital, Shanghai Institute of Maternal-Fetal Medicine and Gynecologic Oncology, Frontier Science Center for Stem Cell Research, School of Life Sciences and Technology, Tongji University, Shanghai 200092, China; Shanghai Key Laboratory of Maternal Fetal Medicine, Clinical and Translational Research Center of Shanghai First Maternity and Infant Hospital, Shanghai Institute of Maternal-Fetal Medicine and Gynecologic Oncology, Frontier Science Center for Stem Cell Research, School of Life Sciences and Technology, Tongji University, Shanghai 200092, China; Shanghai Key Laboratory of Maternal Fetal Medicine, Clinical and Translational Research Center of Shanghai First Maternity and Infant Hospital, Shanghai Institute of Maternal-Fetal Medicine and Gynecologic Oncology, Frontier Science Center for Stem Cell Research, School of Life Sciences and Technology, Tongji University, Shanghai 200092, China; Shanghai Key Laboratory of Maternal Fetal Medicine, Clinical and Translational Research Center of Shanghai First Maternity and Infant Hospital, Shanghai Institute of Maternal-Fetal Medicine and Gynecologic Oncology, Frontier Science Center for Stem Cell Research, School of Life Sciences and Technology, Tongji University, Shanghai 200092, China; Shanghai Key Laboratory of Maternal Fetal Medicine, Clinical and Translational Research Center of Shanghai First Maternity and Infant Hospital, Shanghai Institute of Maternal-Fetal Medicine and Gynecologic Oncology, Frontier Science Center for Stem Cell Research, School of Life Sciences and Technology, Tongji University, Shanghai 200092, China; Shanghai Key Laboratory of Maternal Fetal Medicine, Clinical and Translational Research Center of Shanghai First Maternity and Infant Hospital, Shanghai Institute of Maternal-Fetal Medicine and Gynecologic Oncology, Frontier Science Center for Stem Cell Research, School of Life Sciences and Technology, Tongji University, Shanghai 200092, China; Shanghai Key Laboratory of Maternal Fetal Medicine, Clinical and Translational Research Center of Shanghai First Maternity and Infant Hospital, Shanghai Institute of Maternal-Fetal Medicine and Gynecologic Oncology, Frontier Science Center for Stem Cell Research, School of Life Sciences and Technology, Tongji University, Shanghai 200092, China

**Keywords:** maternal, immune, rebalancing, fetal, interface, SARS

## Abstract

The current coronavirus disease 2019 (COVID-19) pandemic caused by severe acute respiratory syndrome coronavirus (SARS-CoV-2) remains a threat to pregnant women. However, the impact of early pregnancy SARS-CoV-2 infection on the maternal-fetal interface remains poorly understood. Here, we present a comprehensive analysis of single-cell transcriptomics and metabolomics in placental samples infected with SARS-CoV-2 during early pregnancy. Compared to control placentas, SARS-CoV-2 infection elicited immune responses at the maternal-fetal interface and induced metabolic alterations in amino acid and phospholipid profiles during the initial weeks post-infection. However, subsequent immune cell activation and heightened immune tolerance in trophoblast cells established a novel dynamic equilibrium that mitigated the impact on the maternal-fetal interface. Notably, the immune response and metabolic alterations at the maternal-fetal interface exhibited a gradual decline during the second trimester. Our study underscores the adaptive immune tolerance mechanisms and establishment of immunological balance during the first two trimesters following maternal SARS-CoV-2 infection.

## Introduction

Despite the resolution of the coronavirus disease 2019 (COVID-19) pandemic caused by severe acute respiratory syndrome coronavirus 2 (SARS-CoV-2), it remains an ongoing global challenge of paramount societal concern (Que et al., 2023), with significant implications for maternal and fetal health. Previous studies have shown that SARS-CoV-2 infection during pregnancy is associated with fetal complications, including abortion, intrauterine growth restriction, and preterm birth (Dashraath et al., 2020). However, there is still no evidence of vertical transmission of SARS-CoV-2 from mother to fetus (Garcia-Flores et al., 2022). Nevertheless, maternal viral infection has been shown to disrupt the maternal-fetal interface by altering immune cell signaling and potentially leading to long-term implications for fetal development (Abu-Raya et al., 2016). However, there are few reports on the impact of SARS-CoV-2 infection in early pregnancy on the maternal-fetal interface.

The first three months of pregnancy are a critical period for placental development and organogenesis, and the fetus is particularly vulnerable to severe diseases. At this time, trophoblast cells migrate and invade the maternal-fetal interface (Pijnenborg et al., 1980), while decidual immune cells secrete cytokines to maintain immune tolerance and defend against infection (Hanna et al., 2006; Li et al., 2020). The interaction of these cells can promote the development of the placenta and reduce immune rejection of semiallogenic fetal cells (Ashkar and Croy, 1999). Successful pregnancy depends on the balance between immune activation and immune tolerance to embryonic antigens. However, current research on SARS-CoV-2 infection mostly focuses on infection in the third trimester (Chen et al., 2022; Sureshchandra et al., 2022), leaving the effects of such infection on the first and second trimesters unexplored. Hence, it is imperative to comprehensively elucidate the consequences of maternal SARS-CoV-2 infection during the initial stages of pregnancy.

In this study, we investigated the impact of SARS-CoV-2 infection in early pregnancy on the maternal-fetal interface. Single-cell RNA sequencing (scRNA-seq) analysis revealed that SARS-CoV-2 infection during early pregnancy leads to a wide range of antiviral responses in decidual immune cells and placental villous trophoblast cells, as characterized by heightened secretion of type II IFN (IFN-γ) from immune cells and macrophage activation. Upregulation of HLA class I molecule (HLA-G) expression in trophoblast cells subsequently contributes to augmented maternal-fetal immune tolerance and inhibition of the cytokine storm phenomenon. Furthermore, scRNA-seq and metabolomics analyses of placentas at approximately 10–14 weeks post-infection revealed a gradual resolution of the alterations in the maternal-fetal interface induced by SARS-CoV-2 infection as placental development progressed.

## Results

### Characteristics of the study population and single-cell transcriptome profiles of the maternal-fetal interface

**Figure 1.**
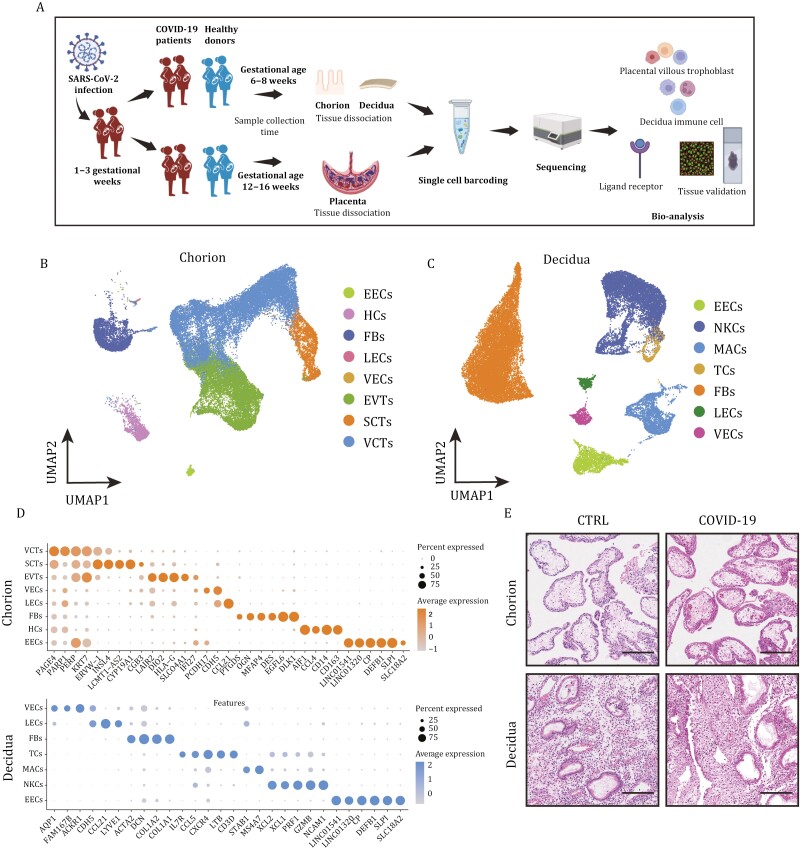
**Characteristics of the study population and single-cell transcriptome profiles of the maternal-fetal interface**. (A) Experimental design for the study. Placentas were collected from healthy donors and SARS-CoV-2-infected pregnancies. The decidua and chorion were separated and processed by mechanical and enzymatic methods to isolate single cells, which were multiplexed and profiled using single-cell RNA sequencing. (B) UMAP plots of first-trimester chorion tissues colored by cell type. (C) UMAP plots of first-trimester decidua tissues colored by cell type. (D) Bubble plots of key gene expression markers used for annotations of cells from first-trimester chorion and decidua tissues. (E) HE staining of decidua and chorion tissues from the first trimester. Scale bar = 200 μm.

A total of 34 pregnant women were recruited as participants for our study (Tables S1 and S2). Those in the infection group were infected with SARS-CoV-2 in December 2022, tested positive for SARS-CoV-2 by RT‒PCR and were in weeks 1 to 3 of the gestational weeks when they were infected. To comprehensively assess the impact of SARS-CoV-2 infection on the development of the maternal-fetal interface, we performed scRNA-seq of placental tissues, including the first-trimester and second-trimester, from pregnant women with SARS-CoV-2 infection and controls (Fig. 1A). After rigorous cell filtration, a total of 84,331 cells with high-quality data were obtained for downstream analyses. We identified eight cell clusters in the chorion (Figs. 1B and S1A) as well as seven cell clusters in the maternal decidua tissues during the 6–8 weeks (first-trimester) (Figs. 1C and S1B). Cluster annotations were determined based on expression of marker genes previously described for first-trimester placental cells (Fig. 1D). Trophoblast clusters included villous cytotrophoblast cells (VCTs) (*PARP1*), syncytiotrophoblasts (SCTs) (*CGB* and *ERVW-1*), and extravillous trophoblasts (EVTs) (*DIO2* and *HLA-G*) (Lee et al., 2016; Suryawanshi et al., 2018; Yi et al., 2023). We also identified immune cell clusters consisted primarily of T cells (TCs), natural killer cells (NKCs), and macrophages (MACs). TCs highly expressed *CCl5*, *LTB* and *CD3D*; NKCs exhibited high expression levels of *XCL2*, *PRF1* and *GZMB*; MACs uniquely expressed *STAB1* and *MS4A7* (Guo et al., 2021; Sureshchandra et al., 2022; Vento-Tormo et al., 2018). Consistent with the HE staining results for various tissues (Fig. 1E), no great differences in cell populations were observed between the infected and uninfected groups, suggesting a lack of discernible tissue alterations following SARS-CoV-2 infection.

### SARS-CoV-2 induces a wide range of antiviral responses at the maternal-fetal interface

**Figure 2.**
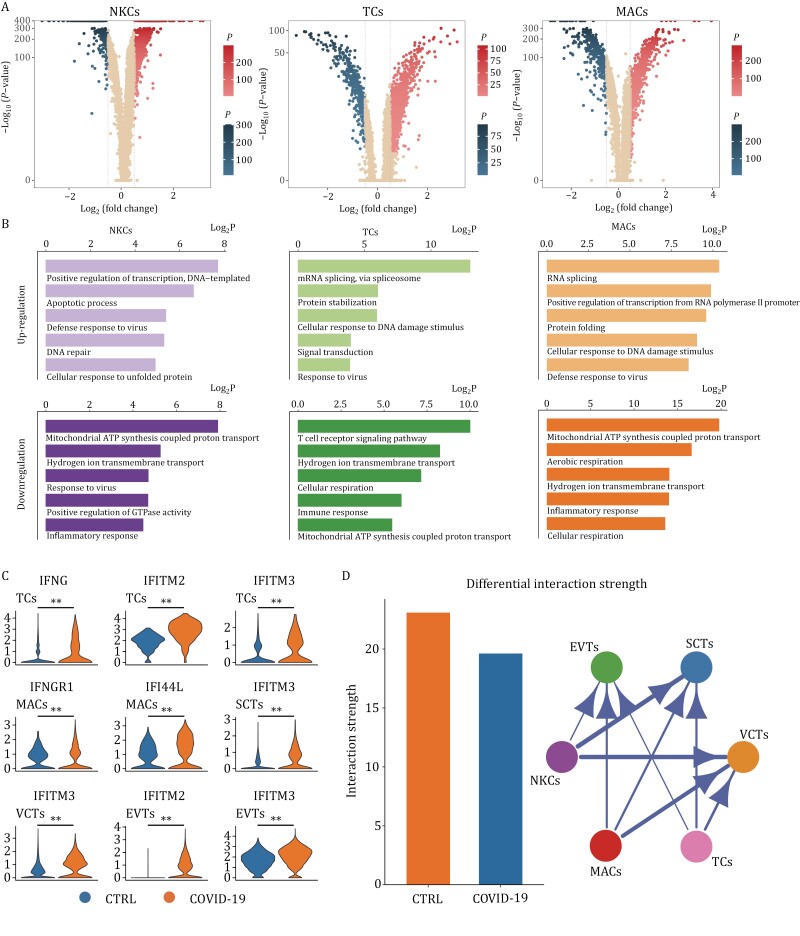
**SARS-CoV-2 induces a wide range of antiviral responses at the maternal-fetal interface**. (A) Volcano plot showing the DEGs in immune cells of decidual tissues from pregnant women infected with SARS-CoV-2. (B) Gene Ontology (GO) enrichment analysis of upregulated and downregulated DEGs in immune cells of decidua tissues from pregnant women infected with SARS-CoV-2. (C) Violin plots of select DEGs in different cell types of the first-trimester control and COVID-19 groups. Expression levels were transformed using logarithm. ** represents *P* < 0.01. (D) The differential interaction strength between immune cells and trophoblast cells. All the interaction strength between specific cells decreased in the COVID-19 group.

To understand changes in the maternal-fetal interface after SARS-CoV-2 infection in pregnant women, we performed differential expression analysis to investigate gene expression changes in decidua immune cells and placental villous trophoblast cells from first-trimester tissues after SARS-CoV-2 infection. There was a marked difference in gene expression levels between the COVID-19 and control groups (Figs. 2A and S2A). In addition, GO analysis based on the DEGs revealed that the upregulated genes in immune cell subsets, including MACs, NKCs, and TCs, as well as in trophoblast cell subsets, including EVTs, SCTs, and VCTs, are all involved in the antiviral response, which indicates that a wide range of antiviral responses occur at the maternal-fetal interface during the first-trimester (Figs. 2B and S2B).

Interferon-induced transmembrane protein (IFITM) family members are responsible for detecting viruses and defending against infection (Bailey et al., 2014). Compared with the control group, levels of *IFITM2* and *IFITM3* in EVTs, *IFITM3* in SCTs and VCTs, *IFNGR1* and *IFI44L* in MACs and *IFNG*, *IFITM2* and *IFITM3* in TCs were significantly upregulated in the COVID-19 group (Fig. 2C), suggesting that a wide range of antiviral responses occur at the maternal-fetal interface after SARS-CoV-2 infection. Furthermore, analysis of cell–cell communication showed a reduction in interaction strength and interaction numbers between immune cell subsets and trophoblast cell subsets in the COVID-19 group (Figs. 2D and S2C). The decline in the incoming interaction strength of immune cells is more evident than the outgoing interaction strength (Fig. S2D). This observation suggests an impact of SARS-CoV-2 infection on the regulation of immune cell behavior toward trophoblast cells.

In conclusion, in comparison to non-infected pregnant women, pregnant women infected with SARS-CoV-2 exhibit aberrant activation of maternal-fetal interface immunity and a broad spectrum of antiviral responses; communication between cells at the maternal-fetal interface is also affected.

### SARS-CoV-2 leads to limited activation of immune cells

Research has demonstrated that the maternal immune system undergoes multiple modifications during early human placental development, which prevent maternal immune cells, particularly NKCs and MACs, from attacking fetal-derived semiallogenic cells in the placenta (Co et al., 2013; Jabrane-Ferrat, 2019). Studies on pregnant women infected with SARS-CoV-2 in late pregnancy have revealed changes in the maternal decidual immune landscape, including loss of tissue-resident decidual MACs and upregulation of MAC cytokine/chemokine signaling, which may have long-term adverse outcomes for the offspring.

**Figure 3.**
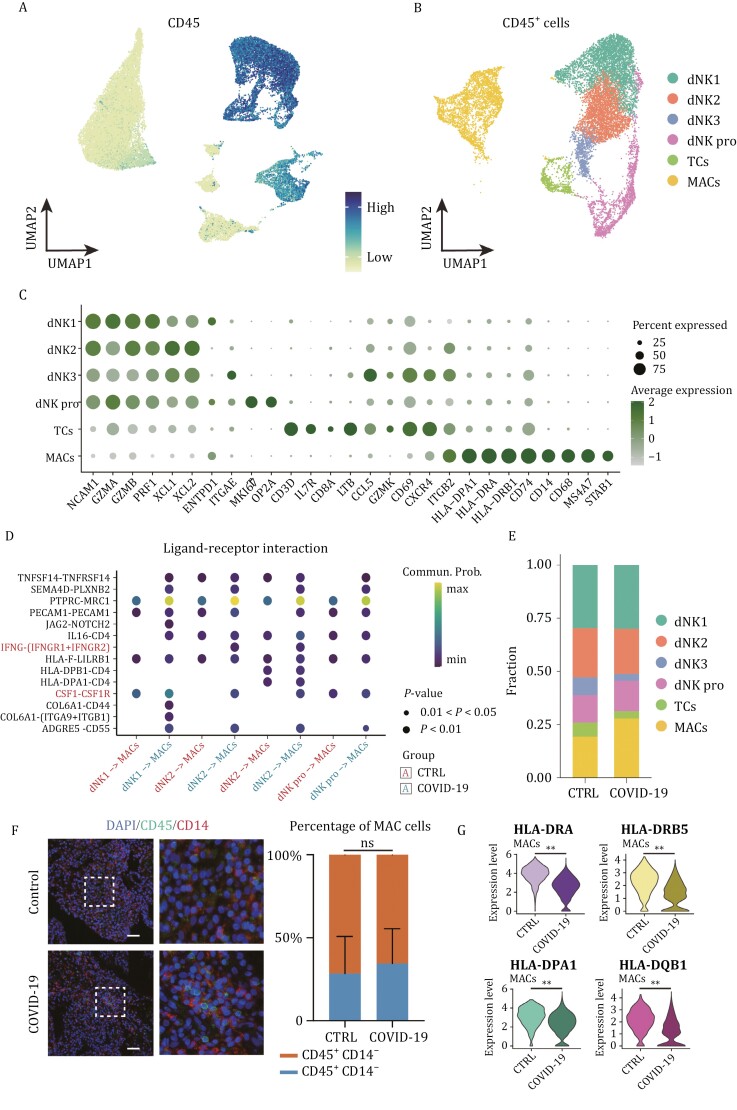
**SARS-CoV-2 leads to limited activation of immune cells.** (A) UMAP plot showing the expression level of CD45 in cells from first-trimester decidua tissues. (B) UMAP plot of CD45^+^ cells from first-trimester decidua tissues. (C) Bubble plots of key gene expression markers used for annotations of immune cells from first-trimester decidua tissues. (D) Bubble plot showing ligand–receptor interactions among NKCs and MACs of the first-trimester control and COVID-19 groups. (E) Stacked bar plot showing percentages of different immune cell types between the first-trimester control and COVID-19 groups. (F) Immunofluorescence staining of CD45 and CD14 (MAC markers) in the decidua (left panel) and a bar plot showing the percentage of MACs between the first-trimester control and COVID-19 groups. Scale bar = 50 μm. (G) Violin plots of select DEGs in MACs of the first-trimester control and COVID-19 groups. Expression levels were transformed using logarithm. ** represents *P* < 0.01.

To further evaluate the specific immune cell populations and their responses to SARS-CoV-2 infection, we classified immune (CD45^+^) cells into subsets and performed dimensional reduction and cell type identification (Fig. 3A–C). Type II IFN (IFN-γ) is mainly produced by immune cells, which play an important role in innate and adaptive immunity against viral, certain bacterial, and protozoan infections and can inhibit SARS-CoV-2 replication by activating macrophages (Ashkar and Croy, 1999; Busnadiego et al., 2020; Xu et al., 2018). Compared with the control group, the expression of IFNG in TCs and that of IFNGR (IFN-γ receptor) in MACs were upregulated in the COVID-19 group (Fig. 2C). In addition, cell–cell communication analyses revealed that IFNG-IFNGR signaling and other macrophage activating factors, such as CSF1 (colony-stimulating Factor 1)-CSF1R (colony-stimulating Factor 1 receptor), between NKCs and MACs were enhanced (Fig. 3D). The above evidence indicates that immune cells at the maternal-fetal interface become activated.

Conversely, the proportion of macrophage-associated cells (MACs) remained unaltered, as demonstrated by immunofluorescence staining of decidual tissues (Fig. 3E and 3F). Expression levels of major histocompatibility complex class II molecules, including HLA-DRA, HLA-DRB5, HLA-DPA1, and HLA-DQB1, which are expected to be increased following MAC activation in the COVID-19 group, were found to decrease (Fig. 3G). Moreover, differentially expressed genes associated with MACs exhibited a downregulation pattern and were significantly enriched in immune response pathways (Fig. 2B). These findings suggest a limited antiviral response at the maternal-fetal interface several weeks following SARS-CoV-2 infection during early pregnancy.

### Immune tolerance in trophoblast cells is enhanced

The phenomenon of maternal-fetal immune tolerance is a multisystem and multifactor balance process of immune activation and inhibition at the maternal-fetal interface (Thellin et al., 2000), among which regulation of HLA class I molecule (HLA-G/HLA-E/HLA-F) expression in fetal trophoblast cells and decidual NK cell activity is most important. NKCs recognize HLA antigens through their surface receptors, transmit inhibitory signals, and regulate NKC cytotoxic activity and cytokine release (Clark et al., 2010; Tang et al., 2011).

**Figure 4.**
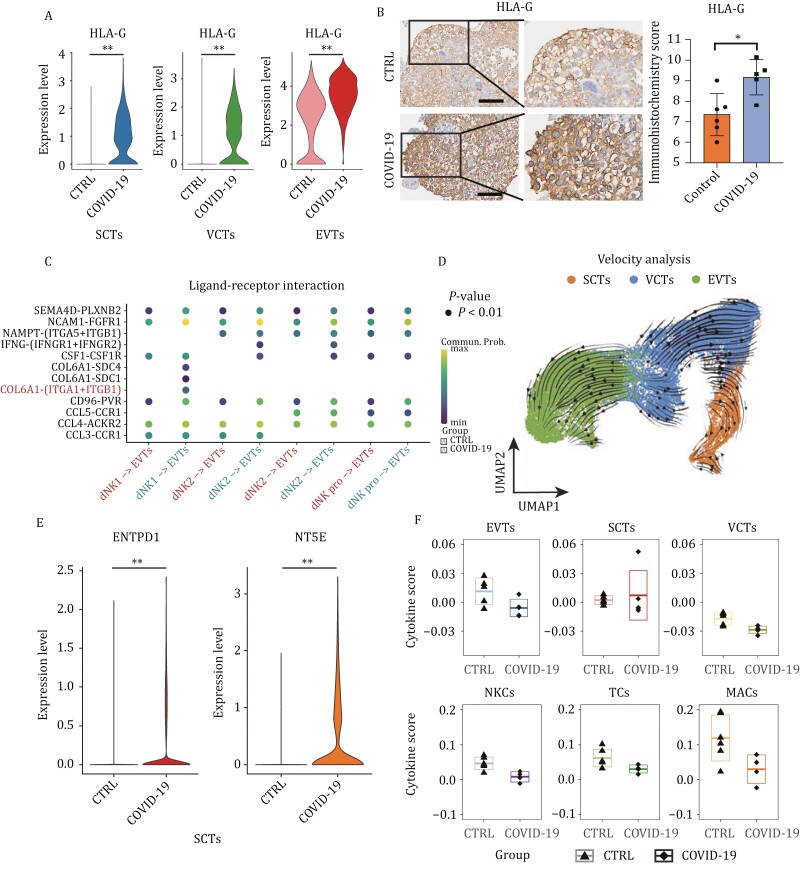
**Immune tolerance in trophoblast cells is enhanced**. (A) Violin plots of HLA-G in trophoblast cells of the first-trimester control and COVID-19 groups. Expression levels were transformed using logarithm. ** represents *P* < 0.01. (B) Immunohistochemistry and the immunohistochemistry score of HLA-G in chorion tissues of the first-trimester control and COVID-19 groups. Scale bar = 100 μm. (C) Bubble plot showing ligand–receptor interactions among NKCs and EVTs of the first-trimester control and COVID-19 groups. (D) RNA velocity streamlines embedded on the UMAP plot of SCTs, EVTs, and VCTs from first-trimester chorion tissues. (E) Violin plots of select DEGs in SCTs of the first-trimester control and COVID-19 groups. Expression levels were transformed using logarithm. ** represents *P* < 0.01. (F) Box plot showing the cytokine scores of different cell types between the control and COVID-19 groups. The mean ± SD of each group is shown in the plot.

HLA-G plays a crucial role in maintaining immune tolerance at the maternal-fetal interface and protecting extravillous trophoblasts (EVTs) from maternal immune cell attack during their invasion into maternal tissues (Chumbley et al., 1994). Our immunohistochemistry analysis confirmed that SARS-CoV-2 infection upregulated HLA-G expression in EVTs, syncytiotrophoblasts (SCTs), and villous cytotrophoblasts (VCTs) (Fig. 4A and 4B). Additionally, enhanced interaction between natural killer cells (NKCs) and EVTs through COL6A1-(ITGA1 + ITGB1) was observed via cell–cell communication analyses, suggesting active invasion and migration of EVTs (Fig. 4C). RNA velocity analysis revealed that the bidirectional differentiation trajectory from VCTs to EVTs and SCTs was unaffected by SARS-CoV-2 infection during the first trimester (Fig. 4D). Notably, immunofluorescence experiments suggest that SARS-CoV-2 infection may alter the proportion of EVT cells, while no significant change was observed in the proportion of SCT cells (Fig. S3A and S3B).

Reduced ATP production under physiological hypoxia has also been reported to inhibit placental inflammatory responses (Vento-Tormo et al., 2018). We observed significant upregulation of CD39 (ENTPD1) and CD73 (NT5E), which was confirmed by immunohistochemical experiments (Fig. S3C), both of which are able to hydrolyze ATP, in SCTs of the COVID-19 group (Fig. 4E). This suggests that the inflammatory response may not exhibit prolonged persistence, potentially due to the immunosuppressive environment at the early maternal-fetal interface and immune tolerance of placental villous cells.

Previous studies have demonstrated that elevated levels of HLA-G can suppress immunoglobulin production and prevent excessive cytokine secretion (Amiot et al., 2014). In this study, we observed the absence of a cytokine storm phenomenon in COVID-19 group cells. Moreover, immune cells exhibited reduced cytokine scores for gene sets associated with cytokine storms (Fig. 4F). These findings are consistent with previous research reporting rare occurrences of cytokine storms in pregnant COVID-19 patients (Hojyo et al., 2020; Wang et al., 2020).

In conclusion, the immune tolerance of trophoblast cells at the maternal-fetal interface increases after SARS-CoV-2 infection, which ensures stability of the maternal-fetal interface. The improved immune tolerance of trophoblast cells may be crucial for preventing excessive immunity-induced cytokine storms and damage to the maternal-fetal interface.

### The impact of SARS-CoV-2 on the maternal-fetal interface gradually diminishes with development

**Figure 5.**
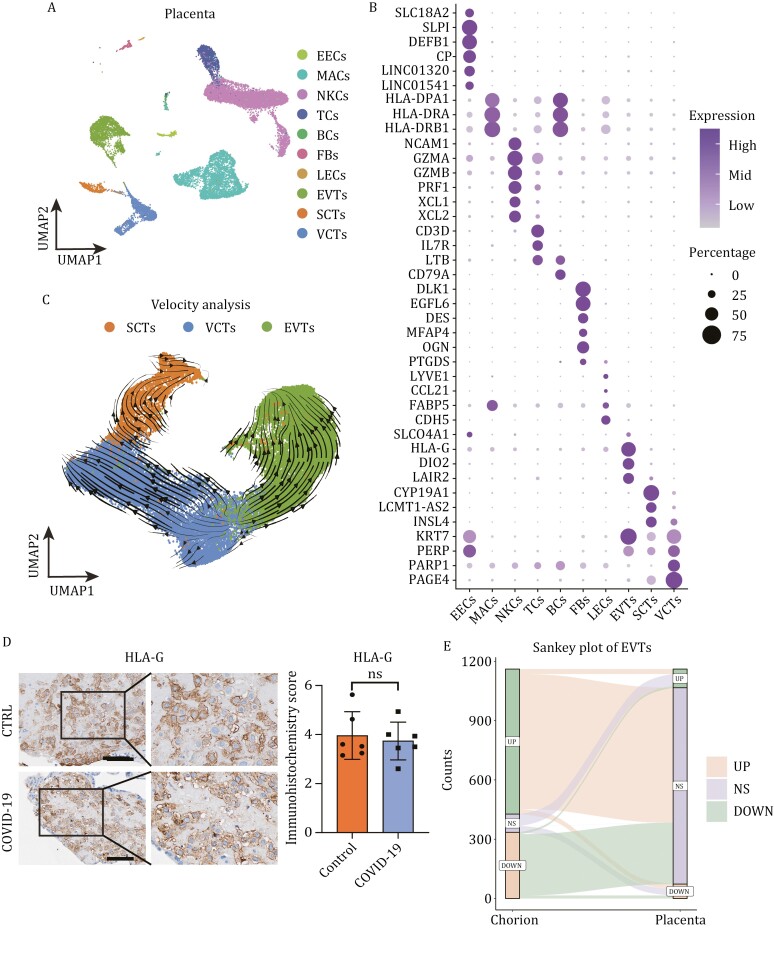
**The impact of SARS-CoV-2 on the maternal-fetal interface gradually diminishes with development.** (A) UMAP plots of second-trimester placental tissues colored by cell type. (B) Bubble plots of key gene expression markers used for annotation of cells from second-trimester placental tissues. (C) RNA velocity streamlines embedded on the UMAP plot of SCTs, EVTs and VCTs from first-trimester chorion tissues and second-trimester placental tissues. (D) Immunohistochemistry and the immunohistochemistry score of HLA-G in the placental tissues of the second-trimester control and COVID-19 groups. Scale bar = 100 μm. (E) Sankey plot showing DEG changes in EVTs between first-trimester and second-trimester tissues. NS means no significance.

Previous studies have found that infection of trophoblast cells with herpes simplex virus (HSV) or cytomegalovirus (CMV) leads to apoptosis and reduced cell invasion (Pereira, 2018; Schust et al., 1996). HPV infection has been associated with aberrant placental development or pregnancy loss (Slatter et al., 2015). To investigate the potential persistent effects of SARS-CoV-2 infection on the maternal-fetal interface, we conducted single-cell sequencing analysis on placental tissues obtained from pregnancies at a gestational age of 12–16 weeks (second-trimester) during miscarriage events (Fig. S4A and S4B). The timing of SARS-CoV-2 infection in these pregnant individuals coincided with the aforementioned period. We identified ten distinct cell types, including trophoblast cells and immune cells (Fig. 5A), while assessing expression levels of markers used for cell identification (Fig. 5B).

EVT invasion and migration to the maternal region is key to ensuring normal development of the placenta. Analysis of the RNA velocity of EVTs from the first trimester to the second trimester revealed a normal differentiation trajectory (Fig. 5C). The immunofluorescence assay revealed no significant difference in the proportion of trophoblast cells between the infected and healthy groups during the second trimester (Fig. S4C and S4D). There was no difference in HLA-G expression between the second-trimester COVID-19 group and the second-trimester control group (Fig. S4E), which was verified by immunohistochemistry (Fig. 5D). Expression levels of chorionic hormone synthesis genes such as *CGB3* and EVT invasion- and differentiation-related genes such as *IGFBP3* and *EGFR* in the second-trimester COVID-19 group were also consistent with those in the second-trimester control group (Fig. S4E). HE staining showed no significant difference in the histological morphology of placental villi (Fig. S4F), suggesting normal EVT differentiation and invasion and chorion development.

In addition, the number of DEGs in cells from the second-trimester groups was significantly lower than that in cells from the first-trimester groups (Fig. S5A). Most of the DEGs identified in the first-trimester groups also became nonsignificant in the second-trimester groups with placental development (Figs. 5E and S5B).

In brief, we found that immune changes at the maternal-fetal interface caused by maternal SARS-CoV-2 infection do not affect cell invasion. There is no evidence that short-term immune responses at the maternal-fetal interface can affect the placental structure. The notable transcriptome disorders observed in cells from the first-trimester COVID-19 group were mitigated as placental tissues developing in the second-trimester stage, indicating a gradual reduction in the impact of SARS-CoV-2.

### The metabolic changes caused by SARS-CoV-2 infection gradually diminish during placental development

**Figure 6.**
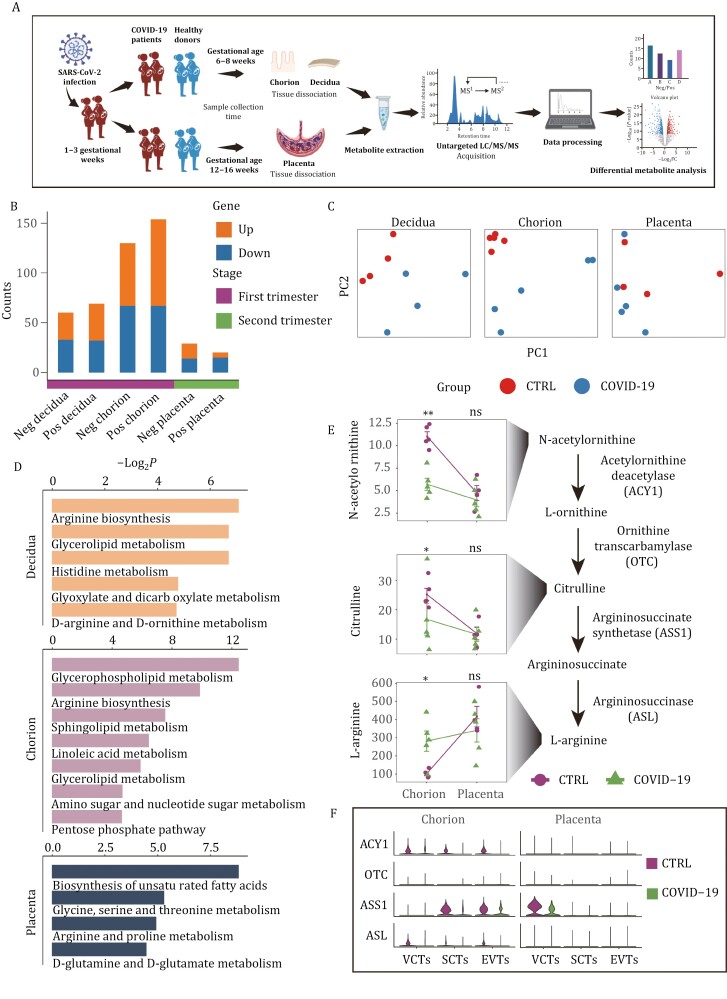
**The metabolic changes caused by SARS-CoV-2 infection gradually diminish during placental development.** (A) Experimental design for untargeted metabolomic analysis. (B) The stacked bar plot showing the counts of differentially regulated metabolites in each group. (C) PCA plots of the samples from three tissues. (D) KEGG analysis of differentially regulated metabolites from three tissues. (E) The diagram of arginine biosynthesis and the normalized levels of some metabolites in control and COVID-19 groups from first-trimester chorion tissues and second-trimester placental tissues. * and ** represent *P* < 0.05 and *P* < 0.01, respectively. (F) Violin plots of genes related to arginine biosynthesis in control and COVID-19 groups from first-trimester chorion tissues and second-trimester placental tissues. Expression levels were transformed using logarithm.

Research has shown that SARS-CoV-2 can also affect the regulation of cellular metabolism (Jia et al., 2022; Masoodi et al., 2022). To investigate this, we collected samples of first-trimester decidua and chorion tissue, as well as second-trimester placenta, to perform untargeted metabolomic analysis (Fig. 6A). All enriched metabolite classes are shown in Fig. S6A. To study differences in metabolite regulation between the normal and COVID-19 groups, we performed differentially abundant metabolite analysis for the metabolites enriched in positive and negative ion modes separately (Fig. S6B). The results revealed that compared to first-trimester decidua and chorion tissues, the number of differentially regulated metabolites was significantly reduced in second-trimester placental tissues (Fig. 6B). This finding suggests a decreasing trend regarding the impact of SARS-CoV-2 on the maternal-fetal interface tissues as pregnancy progresses, which is consistent with our transcriptomic data.

Furthermore, we conducted principal component analysis on all enriched metabolites from different groups of tissues (Fig. 6C) and found that relative to early tissues, the second-trimester placental tissues of the normal and COVID-19 groups exhibited greater similarity and smaller differences, providing further support for the aforementioned observations. Additionally, KEGG analysis of differentially abundant metabolites revealed that the impact of SARS-CoV-2 on maternal-fetal interface cells was mainly concentrated in amino acid metabolism and phospholipid metabolism, which is consistent with previous studies (Adebayo et al., 2021; Masoodi et al., 2022) (Fig. 6D). Notably, our data revealed an elevated level of arginine in the COVID-19 group several weeks post-infection, potentially indicating of an arginine accumulation response to the SARS-CoV-2 infection (Fig. 6E). Additionally, we observed a declining trend in the precursor metabolites and associated gene expressions of the arginine pathway in the COVID-19 group compared to the control group (Fig. 6E and 6F). Furthermore, no significant differences were observed in arginine levels and related gene expressions in placental tissues during the second trimester. Besides arginine, alterations were also detected in some phospholipids levels and their corresponding enzymes within first-trimester chorion tissues; however, these differences became non-significant during the second-trimester placental tissues (Fig. S6C and S6D). In summary, we found that while COVID-19 had an enriched impact on arginine metabolism and phospholipid metabolism 5 weeks post-infection, these effects diminished approximately ten weeks later.

## Discussion

In this study, we investigated the impact of maternal SARS-CoV-2 infection during early pregnancy on the development of the maternal-fetal interface. Single-cell RNA sequencing and metabolomics sequencing were used to describe the impact of SARS-CoV-2 infection during pregnancy on tissue development and the immune landscape. Our experimental observation provides the first direct evidence that abnormal responses to SARS-CoV-2 disappear as the maternal-fetal interface develops. These results suggest that the adaptive immune response at the maternal-fetal interface can limit the impact of SARS-CoV-2 infection, thereby diminishing its effects on placental development. Transcriptome data analysis revealed that the placental cells of pregnant women infected with SARS-CoV-2 in the early stage of pregnancy show a wide range of antiviral responses. However, immune activation at the maternal-fetal interface is limited. We also found that trophoblast cells express higher levels of HLA-G, demonstrating enhanced immune tolerance. Furthermore, the cytokine storm phenomenon caused by excessive secretion of inflammatory factors was not observed. The presence of cytokine storms during the acute phase of infection and their recovery with the progression to sampling are still uncertain. Based on these results, we formulated the following hypothesis: the strong immune tolerance exhibited by trophoblast cells forms a new dynamic equilibrium with limited activation of maternal immune cells. Data show that it is different from SARS-CoV-2 infection in the third trimester; SARS-CoV-2 infection in the early stage of pregnancy did not cause loss of macrophages, and activation of MACs was limited, which may be due to the immunosuppressive environment at the maternal-fetal interface in early pregnancy.

Studies on HSV, HPV, or CMV infection in early pregnancy have found that viral infection may affect placental development, leading to abortion or preterm birth. In this study, placentas were analyzed at different developmental stages after SARS-CoV-2 infection, and it was found that immune activation in the short term after SARS-CoV-2 infection gradually diminishes with placental development. The data support our hypothesis of a new dynamic immune balance at the maternal-fetal interface in the short term due to maternal SARS-CoV-2 infection. In addition, our metabolomics data indicated that the arginine and phospholipid pathways of the COVID-19 group were altered in the first trimester and returned to normal in the second trimester. It has been reported that the elevated arginine level can enhance the metabolic fitness of T cells and promote T cell survival (Geiger et al., 2016; Shen and Wang, 2021), and COVID-19 patients showed altered phospholipid levels, which is associated with COVID-19 severity (Shen et al., 2020; Wei et al., 2023). The expression levels of enzymes involved in arginine biosynthesis also exhibited downregulation in COVID-19 groups compared to the control groups, suggesting a potential feedback regulation of arginine that requires further exploration.

Our data provide a reference for clinical and public concerns about the long-term effects of SARS-CoV-2 infection on maternal-fetal interface development in pregnant women. Existing studies have primarily focused on women infected with SARS-CoV-2 in late pregnancy, resulting in a gap in research regarding infection in early pregnancy, which our data help to address. However, the impact of SARS-CoV-2 infection on the maternal-fetal interface over a longer period of time still needs to be explored (Chen et al., 2023). In addition, this study has certain limitations, as it was specific to pregnant women who were infected with the Omicron variant in Shanghai in December 2022. Further studies are needed for other SARS-CoV-2 variants. Due to differences in age and underlying health conditions, the immune response of individuals varies greatly after SARS-CoV-2 infection, and irreversible damage to the maternal-fetal interface cannot be ruled out in some pregnant women.

## Experimental models and subject details

### Tissue collection

Human placental samples were obtained immediately within min of delivery at Shanghai First Maternity and Infant Hospital. Informed consent was obtained from all enrolled subjects. All participants in this study were healthy and did not report any significant comorbidities or complications with pregnancy. The pregnant women were infected with SARS-CoV-2 during their 1–3 weeks of pregnancy. There were 10 cases in the SARS-CoV-2 infection group and 5 cases in the control group, with gestational ages of 6–8 weeks (first trimester), and 11 cases in the COVID-19 group and 8 cases in the control group, with gestational ages of 12–16 weeks (second trimester). The maternal clinical information is shown in Tables S1 and S2. Placentas, including the chorion and decidua basalis (maternal membrane), were obtained from pregnant women without any illnesses. The collected tissues were placed in phosphate-buffered saline (PBS) containing 0.04% bovine serum albumin (BSA) (SIGMA A1470) and stored at 4°C for transport.

### Mechanical dissociation and enzymatic digestion

The tissue was washed by immersing it in 15 mL of PBS in a 10-cm Petri dish to remove excess blood. Next, we weighed 2–3 g of the tissue using an analytical balance and transferred it into a prepared dissociation solution: DMEM/F12 (GIBCO 11320-033) containing 0.1% collagenase A (SIGMA 10103586001). Subsequently, we used a pair of sharp dissecting scissors to mince the tissue into small pieces in a dissociation solution. The minced samples were then incubated in a water bath preheated to 37°C for 30 min, with gentle inversion every 10 min to ensure complete digestion.

### Cell filtration and erythrocyte lysis

The dissociated tissue was filtered through a 100-µm cell strainer (BIOFIL CSS013100) into a 50-mL centrifuge tube. The tube was then centrifuged at 1000 rpm for 5 min at 4°C. Afterward, the supernatant was carefully aspirated without disturbing the pellet. The pellet was resuspended in 5 mL of Red Cell Lysis Buffer (TIANGEN RT122-02), and the cell suspension was incubated for 3–5 min at room temperature with tapping every minute to prevent the suspension from settling during lysis. The reaction was stopped by adding 0.04% BSA solution to a final volume of 50 mL in a centrifuge tube. The tube was then centrifuged at 1000 rpm for 5 min at 4°C. Again, the supernatant was aspirated without disturbing the pellet, which was gently resuspended in 1 mL of 0.04% BSA solution; the cell suspension was filtered through a 40-µm cell strainer (BIOFIL CSS01340) into a 15-mL centrifuge tube.

### Identification of cell viability and removal of dead cells

Cell viability after filtration and concentrations of total, live, and dead cells were recorded. If the cell viability was less than 80%, dead cells were removed using Dead Cell Removal Kit (Miltenyi Biotec 130-090-101).

### Single-cell RNA sequencing

DNBelab C Series Single-Cell Library Prep Set (MGI) was utilized as previously described (Liu et al., 2019) for single-cell RNA-seq library preparation. In brief, single-cell suspensions were converted to barcoded scRNA-seq libraries through steps including droplet encapsulation, emulsion breakage, mRNA-captured bead collection, reverse transcription, cDNA amplification, and purification. Indexed sequencing libraries were constructed according to the manufacturer’s protocol. Concentrations were measured with a Qubit ssDNA Assay Kit (Thermo Fisher Scientific, Q10212), and the libraries were sequenced using a DNBSEQ-T7 sequencer with the following sequencing strategy: 30-bp read length for read 1 and 100-bp read length for read 2.

### Quality control, mapping, and dimensional reduction of scRNA-seq

To obtain the gene expression matrix, fastq files were analyzed using either cellranger v7.1.0 or DNBC4tools v2.0.7. The control group fastq files of the first-trimester were downloaded from ArrayExpress (E-MTAB-6701) (Vento-Tormo et al., 2018). The R package “DoubletFinder” was utilized to filter out doublets (McGinnis et al., 2019). Subsequently, the scRNA-seq data for the control and COVID-19 groups were normalized and integrated using the “Seurat” package by following standard pipelines (Butler et al., 2018). Dimensional reduction was achieved through uniform manifold approximation and projection (UMAP) (McInnes et al., 2018).

### scRNA-seq analysis

To identify DEGs between the control and COVID-19 groups, the “FindMarkers” function from the “Seurat” package was employed. Genes with a *P* value less than 0.05 and a log_2_(fold change) greater than 0.5 were considered DEGs. The upregulated and downregulated genes were separately analyzed using Metascape to perform GO analysis by following the provided instructions (Zhou et al., 2019). For the cytokine score, the “AddModuleScore” function from the “Seurat” package was applied, and the gene set was downloaded from Molecular Signatures Database (Liberzon et al., 2015). To infer cell‒cell communication between different cell types, the R package “CellChat” was utilized by following the standard pipeline (Jin et al., 2021).

### RNA velocity analysis

For RNA velocity analysis, spliced and unspliced counts were obtained by the software “velocyto” from fastq files(La Manno et al., 2018). RNA velocity was calculated based on a likelihood-based dynamical model by following the standard pipeline via the package “scVelo” (Bergen et al., 2020). The RNA velocity streamlines were embedded on the UMAP plot obtained from the “Seurat” package.

### Metabolomics analysis

Metabolomics analysis was conducted by Shanghai Applied Protein Technology Co., Ltd. Briefly, metabolites from samples were extracted through lysis, sonication, and centrifugation. Untargeted metabolomics of polar metabolites was performed, and extracts were analyzed using a quadrupole time-of-flight mass spectrometer (Sciex TripleTOF 6600) coupled to hydrophilic interaction chromatography via electrospray ionization. The mass spectrometer was operated in both negative ion and positive ion modes.

The raw MS data were converted to MzXML files using ProteoWizard MSConvert before data processing using XCMS software. In the extracted ion features, only variables having more than 50% of the nonzero measurement values in at least one group were retained. After normalization to total peak intensity, differentially expressed metabolite analysis was performed, and metabolites with log_2_(fold change) > 1 and *P* value < 0.05 were considered differentially expressed metabolites. Differentially expressed metabolites were then uploaded to MetaboAnalyst 5.0 for KEGG pathway annotation. Pathways with *P* values < 0.05 were considered significantly changed pathways.

### HE and immunostaining

For hematoxylin and eosin (HE) staining and immunohistochemistry (IHC), tissue samples were fixed with 10% buffered formalin at room temperature for 6 h. Subsequently, the samples were embedded in paraffin. Following routine rehydration and antigen retrieval, 5-μm paraffin sections were stained with HE or the anti-HLA-G primary antibody (Beyotime AG8439). The sections were incubated with HRP-conjugated secondary antibody and visualized using a DAB (MXB KIT-5920) solution containing 0.03% H_2_O_2_. The sections were counterstained with hematoxylin and mounted.

For immunofluorescence (IF), CD45 (Servicebio GB14038) and CD14 (Servicebio GB113374) costaining were performed. Paraffin sections were dewaxed for antigen retrieval, and the tissue areas were circled with an IHC pen before blocking for 30 min. Following blocking, primary antibodies were drop-applied, and the slides were placed in a wet box at 4°C overnight. Subsequently, the slides were washed three times with PBS (pH 7.4), and the secondary antibody (Servicebio GB25301/GB25303) was added. Incubation at room temperature in the dark continued for 50 min, after which the slides were washed, and the nuclei were counterstained with DAPI staining solution. An anti-fluorescence quenching sealing agent was applied to reduce tissue autofluorescence, and the slides were sealed with a coverslip.

## Supplementary Material

pwae006_suppl_Supplementary_Materials

## Data Availability

All data associated with this study are presented in the paper or the Supplementary Materials. The sequencing data used in this article are available in ArrayExpress and Genome Sequence Archive for Human of the China National Center for Bioinformation and can be accessed with E-MTAB-6701 and HRA005470.

## Code availability

All the custom codes are available from the corresponding authors upon reasonable request. All used software is publicly available.
