# Supplementary material for: Immune rebalancing at the maternal-fetal interface of maternal SARS-CoV-2 infection during early pregnancy
Source: Protein Cell. 2024 Mar 5;15(6):460–73. doi: 10.1093/procel/pwae006 (PMC11131034; doi:10.1093/procel/pwae006)
Supplement: pwae006_suppl_Supplementary_Materials [file pwae006_suppl_supplementary_materials.pdf]

Supplementary materials

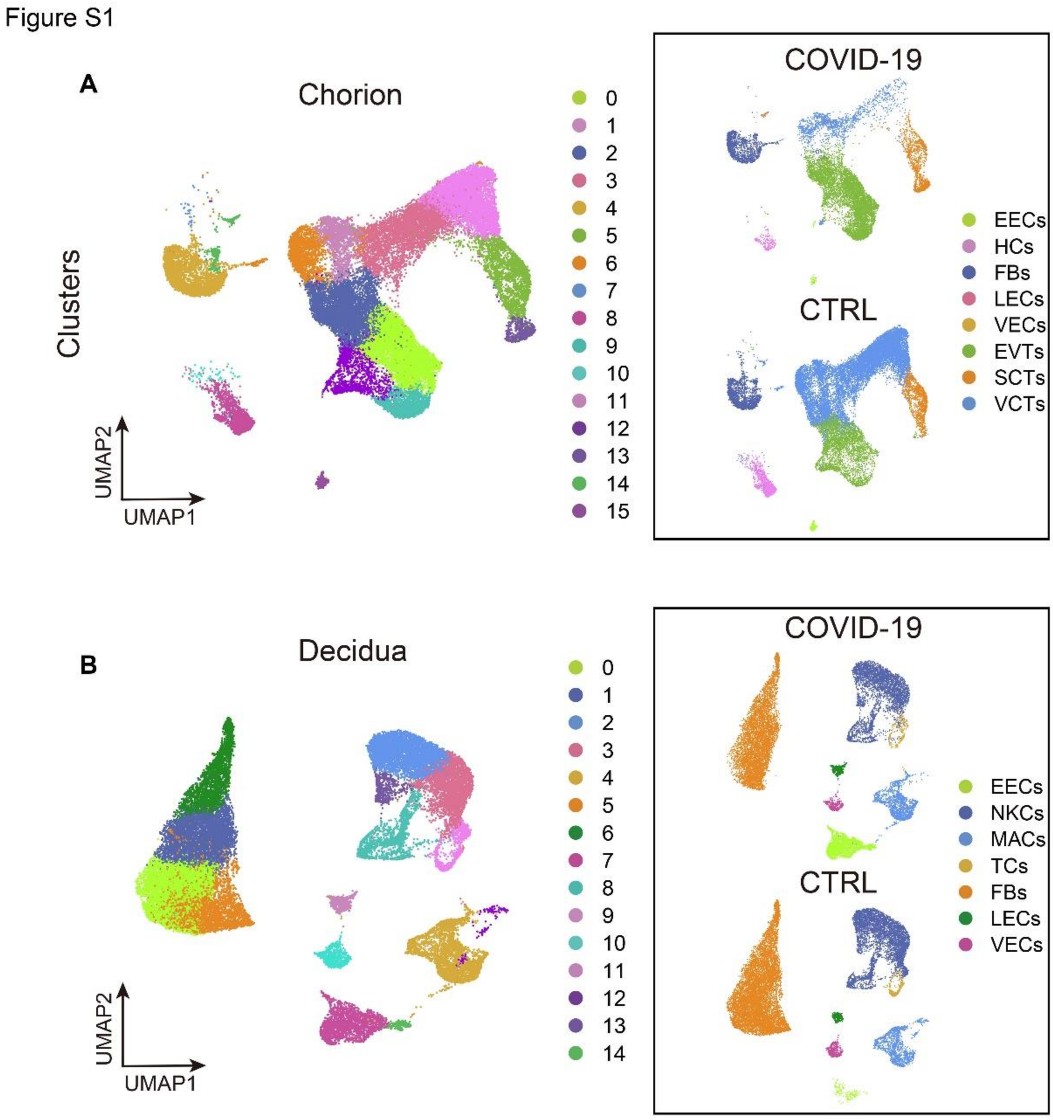

FigureS1. Characteristics of the study population and single-cell transcriptome profiles of the maternal-fetal interface. (A) UMAP plots of first-trimester chorion tissues colored by seurat clusters (left panel) and cell types (right panel). In the right panel, the UMAP plots are split according to the groups. (B) UMAP plots of first-trimester decidua tissues colored by seurat clusters (left panel) and cell types (right panel). In the right panel, the UMAP plots are split according to the groups.

Figure S2

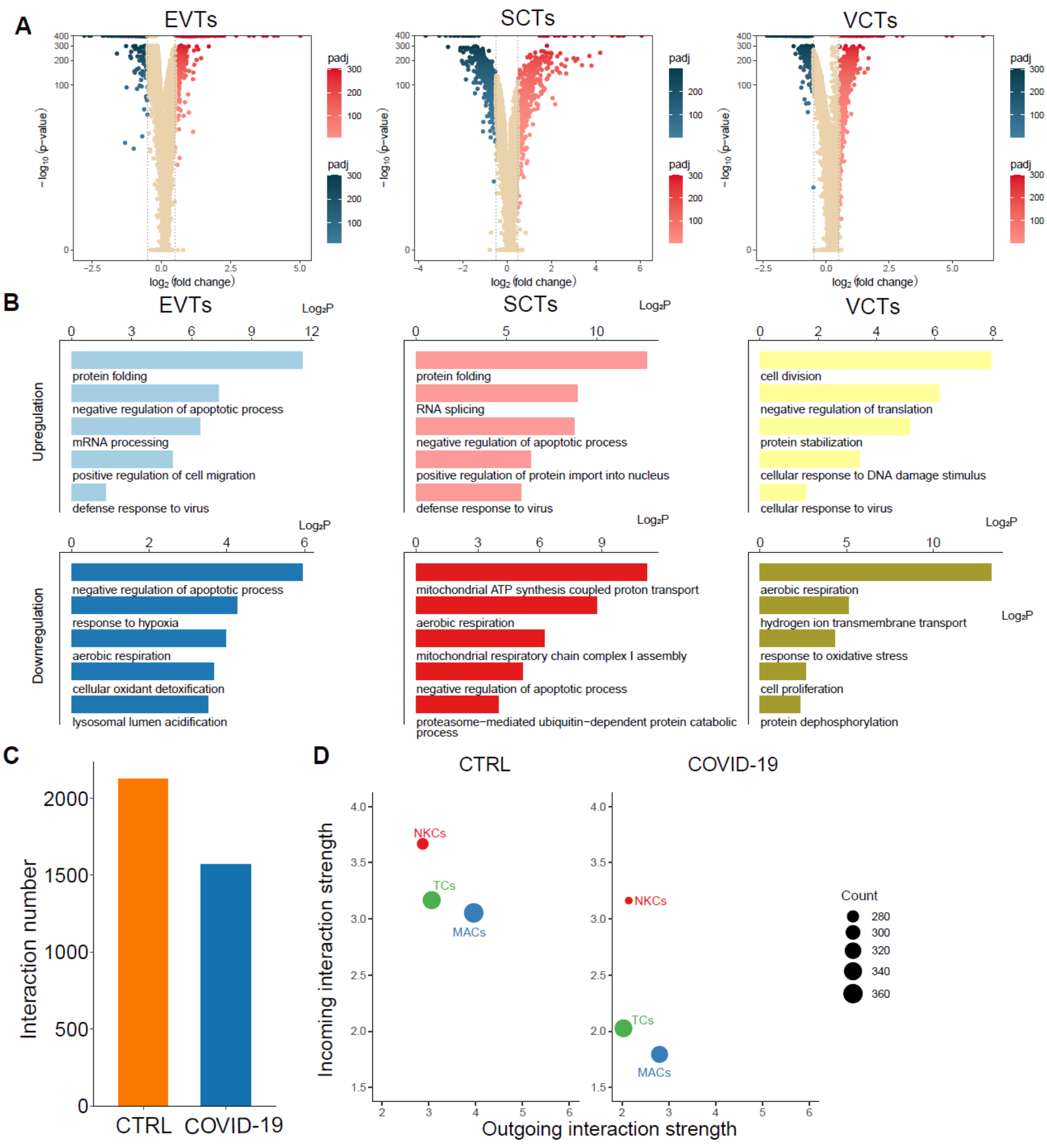

FigureS2. SARS-CoV-2 induces a wide range of antiviral responses at the maternal-fetal interface. (A) The volcano plot showing the DEGs in trophoblast cells of chorion tissues caused by pregnant women infected with SARS-CoV-2. (B) Gene Ontology (GO) enrichment analysis of upregulated and downregulated DEGs in trophoblast cells of chorion tissues caused by pregnant women infected with SARS-CoV-2. (C) The differential interaction number between immune cells and trophoblast cells. (D) The incoming and outgoing interaction strength of three immune cells.

Figure S3

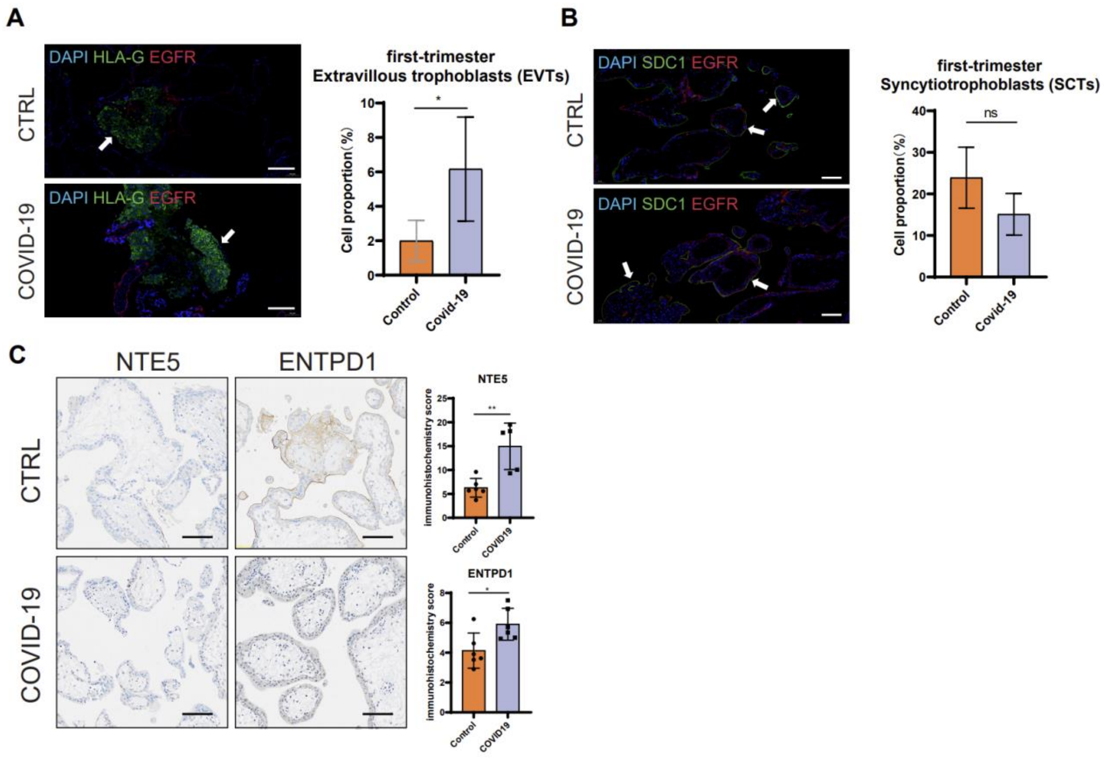

FigureS3. Immune tolerance in trophoblast cells is enhanced. (A) Immunofluorescence staining of EGFR and HLA-G (EVT markers) in the chorion and a bar plot showing the percentage of EVTs between the first-trimester control and COVID-19 groups. Scale bar = 200 μm, n=3(Samples from three pregnant women). (B) Immunofluorescence staining of EGFR and SDC1 (SCT markers) in the chorion and a bar plot showing the percentage of SCTs between the first-trimester control and COVID-19 groups. Scale bar = 100 μm, n=3(Samples from three pregnant women). **(C)** Immunohistochemistry and the immunohistochemistry score of NTE5 and ENTPD1 in chorion tissues of the first-trimester control and COVID-19 groups. Scale bar = 100 μm, n=5.

Figure S4

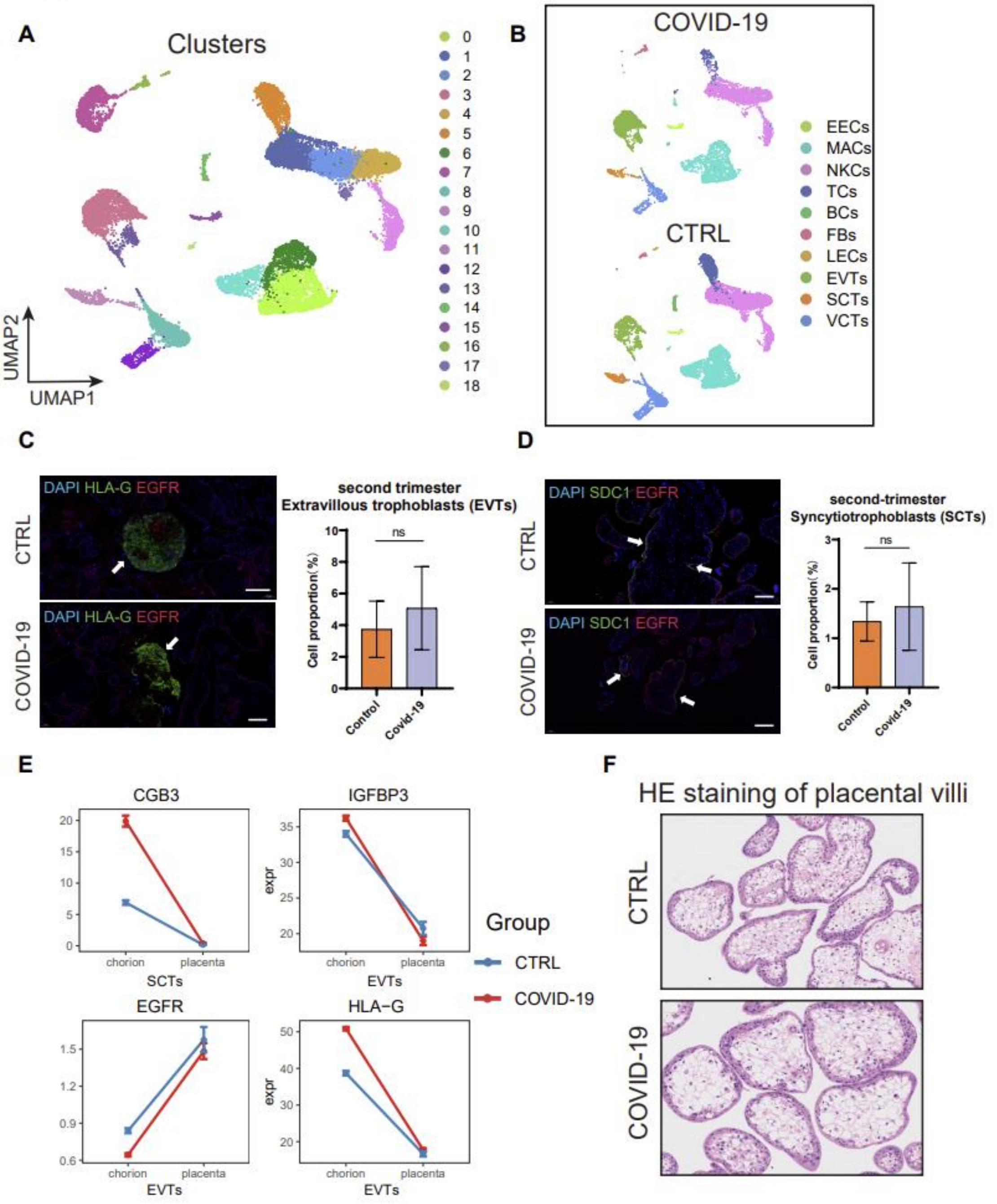

FigureS4. The impact of SARS-CoV-2 on the maternal-fetal interface gradually diminishes with development. (A) The UMAP plot of second-trimester placenta tissues colored by seurat clusters. (B) UMAP plots of second-trimester placenta tissues colored by cell types. The UMAP plots are split according to the groups. (C) Immunofluorescence staining of EGFR and HLA-G (EVT markers) in the placental and a bar plot showing the percentage of EVTs between the second-trimester control and COVID-19 groups. Scale bar = 200 μm, n=3(Samples from three pregnant women). (D) Immunofluorescence staining of EGFR and SDC1 (SCT markers) in the placental and a bar plot showing the percentage of SCTs between the second -trimester control and COVID-19 groups. Scale bar = 100 μm, n=3(Samples from three pregnant women). (E) Trends of DEGs in different cell types from first-trimester to second-trimester. (F) HE staining of placental villi from the second trimester.

Figure S5

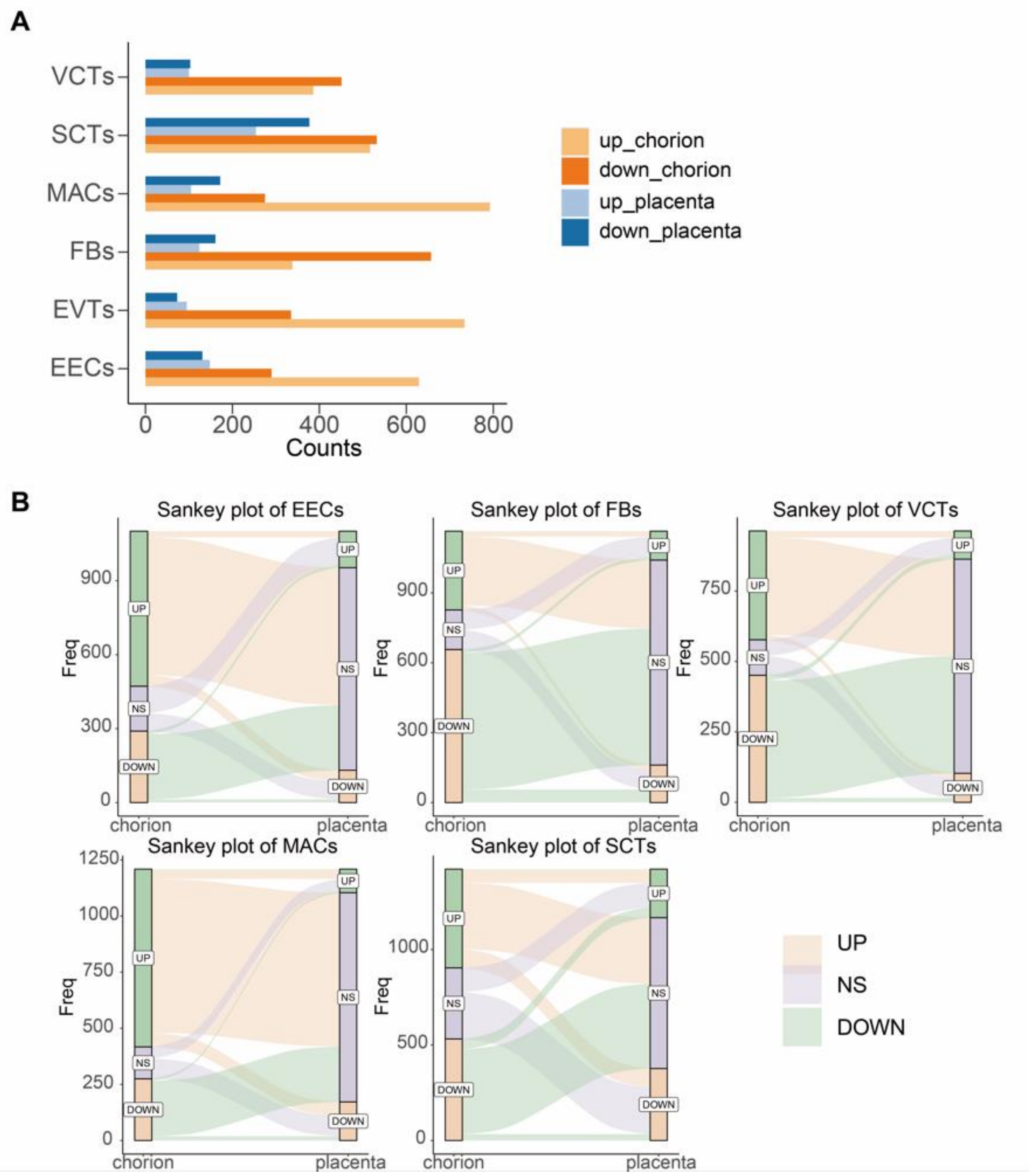

FigureS5. The number of significant upregulated and downregulated DEGs between the control and COVID-19 groups gradually reduced. (A) The stacked bar plot showing the number of significant upregulated and downregulated DEGs in the same cell type of first-trimester and second-trimester tissues. (B) Sankey plot showing the DEG changes in different cell types between first-trimester and second-trimester tissues. NS means no significance.

Figure S6

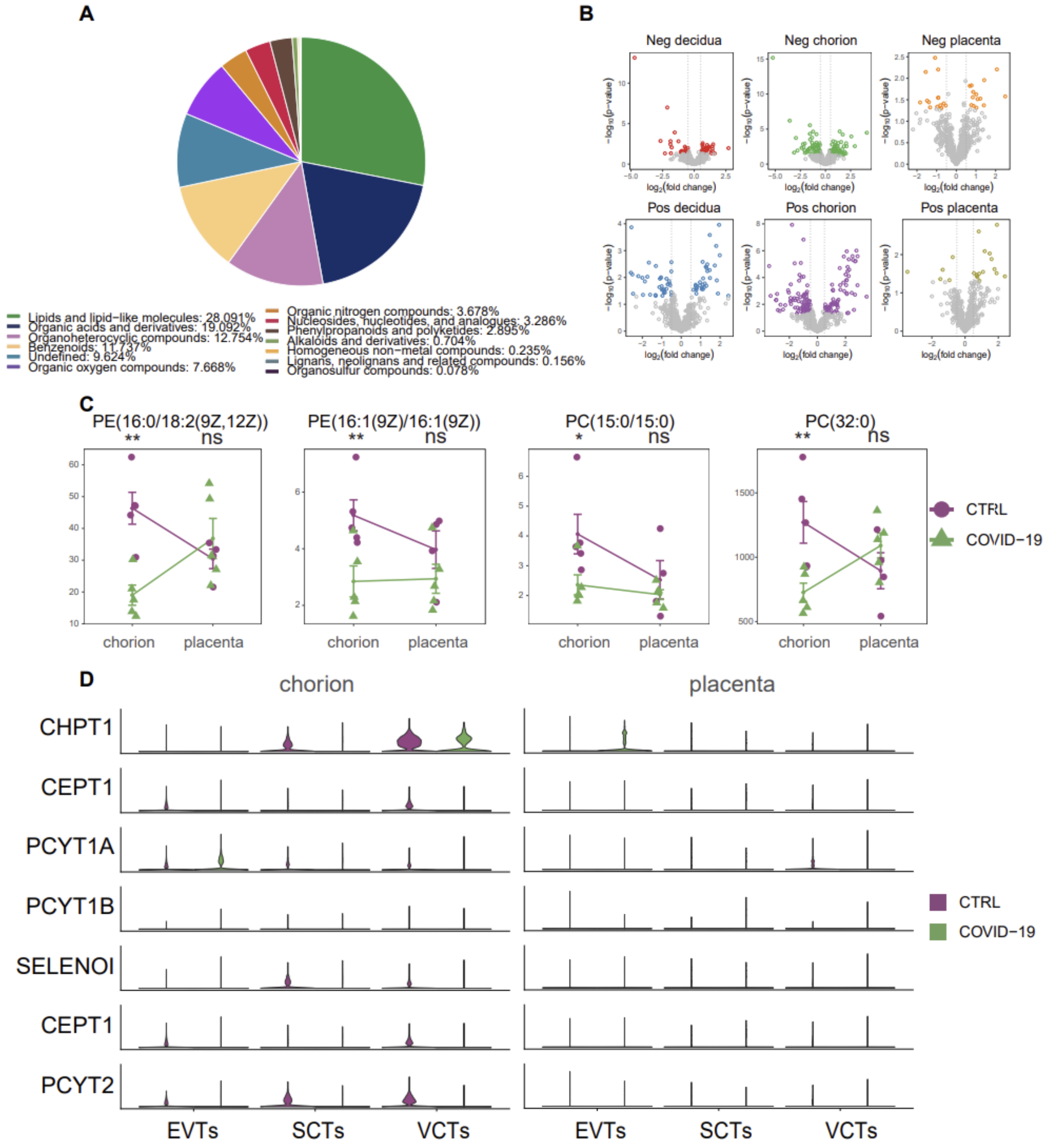

FigureS6. The metabolic changes caused by SARS-CoV-2 infection gradually diminish during placental development. (A) Pie plot showing the proportion of annotated metabolites within each class. (B) Volcano plots showing upregulated and downregulated metabolites enriched in different tissues using positive and negative ion modes. (C) The normalized levels of various types of phospholipids in CTRL and COVID-19 groups from first-trimester chorion tissues and second-trimester placental tissues. * and ** represent $p < 0.05$ and $p < 0.01$, respectively. (D) Violin plots of genes related to phospholipid biosynthesis in CTRL and COVID-19 groups from first-trimester chorion tissues and second-trimester placental tissues. Expression levels were transformed using logarithm.

Table S1. The maternal clinical information

	Chorion of the first trimester (COVID-19 group)	Decidua of the first trimester (COVID-19 group)	Placenta of the second trimester (COVID-19 group)	Placenta of the second trimester (CTRL group)
**Maternal Age (mean)**	**26-34 (28.2)**	**26-33 (26.75)**	**26-38 (32)**	**32-36 (34)**
**Gestational age at elective termination(week)**	**6-8W**	**6-8W**	**12-16W**	**12-16W**
**Days of gestation by LMP (day)**	**55.4 ±5.1**	**54.5 ±5.3**	**95.7 ±10.7**	**100.5 ±6.7**
**Systolic blood pressure,mmHg (mean)**	**87-108 (99.4)**	**94-108 (102.5)**	**95-122 (110.5)**	**86-141 (107)**
**Diastolic blood pressure,mmHg (mean)**	**53-71 (65.6)**	**64-71 (68.75)**	**69-82 (74.3)**	**64-75 (69.5)**
**PLT,10^{9}/L (mean)**	**137-406 (248)**	**200-410 (275.75)**	**191-287 (221.7)**	**180-242 (212.5)**
**WBC,10^{9}/L (mean)**	**4.521-10.79 (7.2042)**	**6.22-10.79 (7.875)**	**5.54-11.67 (8.116)**	**5.9-10.91 (8.225)**
**HGB,g/L (mean)**	**121-142 (128.6)**	**124-142 (130.5)**	**97-128 (118.33)**	**101-114 (106)**
**HCG,mIU/Ml (mean)**	**21000-126248 (48956.88)**	**22650-113529 (55945.48)**	**57492-153622 (99592)**	**56193-145844 (98388)**
**Maternal chronic health conditions**	**NO**	**NO**	**NO**	**NO**
**Cell viability (%)**	**81.4% ±4.1%**	**81.1% ±5.8%**	**89.0% ±2.8%**	**87.0% ±3.2%**

Table S2. Details of clinical characteristics of subjects included in the study

Group	Sample ID	Gestational age at elective termination(weeks)	SARS-CoV-2 infection	Fever	Maximum fever temperature (℃)	Duration of fever(days)	Gestational age at SARS-CoV-2 infection(weeks)	Vaccination status(does)
CTRL	C1	6	no	---	---	---	---	1
	C2	6	no	---	---	---	---	0
	C3	7	no	---	---	---	---	2
	C4	8	no	---	---	---	---	2
	C5	7	no	---	---	---	---	0
	C6	12	no	---	---	---	---	3
	C7	13	no	---	---	---	---	2
	C8	12	no	---	---	---	---	0
	C9	16	no	---	---	---	---	1
	C10	13	no	---	---	---	---	1
	C11	13	no	---	---	---	---	3
	C12	14	no	---	---	---	---	2
	C13	16	no	---	---	---	---	2
Covid-19	S1	8	yes	no	---	---	2	0
	S2	8	yes	yes	38	2	3	0
	S3	7	yes	yes	38.5	3	1	1
	S4	6	yes	yes	39	3	1	2
	S5	7	yes	yes	38.7	3	2	2
	S6	6	yes	no	---	---	3	2
	S7	6	yes	yes	37.8	2	1	1
	S8	8	yes	yes	38	4	2	2
	S9	6	yes	yes	38	2	1	0
	S10	7	yes	yes	38.5	1	3	1
	S11	12	yes	no	---	---	2	3
	S12	15	yes	yes	38.5	3	3	2
	S13	12	yes	yes	39.2	2	2	3
	S14	13	yes	yes	39	3	2	0
	S15	12	yes	yes	38.7	3-4	1	1
	S16	15	yes	yes	38.5	2	1	2
	S17	13	yes	no	---	---	3	2
	S18	15	yes	yes	37.9	2-3	2	0
	S19	12	yes	yes	39	3	1	2
	S20	15	yes	yes	38	3	1	2
	S21	14	yes	yes	39.3	2	2	1
